# Comparison of the Montreal Cognitive Assessment and the Mini‐Mental State Examination in community‐dwelling very old Blacks/African Americans

**DOI:** 10.1002/alz70860_103916

**Published:** 2025-12-23

**Authors:** Elizabeth Guerrero‐Berroa, Kim Love, Ruby S.C. Phillips, Vahram Haroutunian

**Affiliations:** ^1^ Lehman College, City University of New York, Bronx, NY, USA; ^2^ Icahn School of Medicine at Mount Sinai, New York, NY, USA; ^3^ K.R. Love Quantitative Consulting and Collaboration, Athens, GA, USA; ^4^ James J. Peters VA Medical Center, New York, NY, USA; ^5^ Alzheimer's Disease Research Center, Icahn School of Medicine at Mount Sinai, New York, NY, USA; ^6^ Nash Family Department of Neuroscience, Icahn School of Medicine at Mount Sinai, New York, NY, USA

## Abstract

**Background:**

The Montreal Cognitive Assessment (MoCA) is becoming the most widely used screening instrument for cognitive impairment and dementia. However, in underrepresented/underserved populations such as African Americans, MoCA performance can lead to high rates of false‐positive results and consequently misdiagnoses. The literature comparing the MoCA to the Mini‐Mental State Examination (MMSE) is scarce, especially in community‐dwelling very old African Americans. The MoCA has been found more effective in identifying cognitive impairment, especially among those with high educational attainment. However, for African Americans, studies have rarely compared the MoCA and the MMSE. In this population, we examined MoCA performance and compared it to the MMSE.

**Methods:**

Participants were 31 community‐dwelling, very old adults, without dementia, who self‐identified as Blacks/African Americans. Of these, 29 had complete demographics, MoCA, and MMSE data. Mean, SD, and range of MoCA and MMSE, and their intercorrelation, are provided after adjustment for demographics (age and education). Unadjusted correlations with demographics are also presented.

**Results:**

Participants had age mean = 81.7, *SD* = 4.5, range=75‐93; educational attainment mean = 14.6, *SD* = 4.0, range=7‐22, 17.2% < 12; 86.2% were women. The MoCA score had mean = 22.2, *SD* = 4.0, range=14‐27; MMSE score had mean = 27.2, *SD* = 2.1, range = 22‐30. Among those with educational attainment below high school (*n* = 5), MoCA score had mean = 15.6, SD=1.5, median = 15.0, range=14‐18; and MMSE score had mean = 25.0, SD = 2.3, median = 26, range=22‐27. While higher MoCA score was significantly associated with younger age (*r* = ‐.406, *p* = .029) and higher education (*r* = .496, *p* = .006), MMSE associations were nonsignificant for age and education (*r* = ‐.313, *p* = .098; r=.328, *p* = .083, respectively). MoCA and MMSE were significantly associated (partial r=.425, *p* = .027).

**Conclusions:**

Consistent with previous findings in young‐old Blacks/African Americans compared to their White counterparts, our very old sample showed poorer performance on the MoCA compared to the MMSE. These results were strongest among those who did not complete a high school degree. In this population, false‐positive results can be minimized with careful interpretation of both MoCA and MMSE performance. Due to the small sample, these results should be interpreted with caution. However, they do suggest that, until better screening tools are developed for this population, researchers and clinicians should consider the use of the MMSE.

